# Trends in incidence, mortality, and causes of death associated with systemic sclerosis in Denmark between 1995 and 2015: a nationwide cohort study

**DOI:** 10.1186/s41927-018-0043-6

**Published:** 2018-12-07

**Authors:** Sheraz A. Butt, Jørgen L. Jeppesen, Christine Fuchs, Mette Mogensen, Merete Engelhart, Christian Torp-Pedersen, Gunnar H. Gislason, Søren Jacobsen, Charlotte Andersson

**Affiliations:** 1 Department of Internal Medicine and Cardiology, Amager Hvidovre, Glostrup Hospital, Valdemar Hansensvej 1-23, 2600 Glostrup, Denmark; 2 Department of Dermatology, Bispebjerg Hospital, Bispebjerg Bakke 23, 2400 Copenhagen, Denmark; 3 Department of Rheumatology, Herlev and Gentofte Hospital, Gentofte, Kildegårdsvej 28, 2900 Hellerup, Denmark; 4 Department of Health Science and Technology, Aalborg University, Fredrik Bajers Vej 7, 9220 Aalborg, Denmark; 5 Department of Cardiology, Herlev and Gentofte Hospital, Gentofte, Kildegårdsvej 28, 2900 Hellerup, Denmark; 6 Copenhagen Lupus and Vasculitis Clinic, Rigshospitalet, Blegdamsvej 9, 2100 Copenhagen, Denmark

**Keywords:** Systemic sclerosis, Scleroderma, Incidence, Mortality, Cause of death, Epidemiology

## Abstract

**Background:**

To investigate the incidence and the mortality-rates of systemic sclerosis (SSc), its primary causes of death, and the temporal trends in events in Denmark during the last decades.

**Methods:**

Using the Danish National Patient Registry, we identified all persons aged ≥18 years with a first-time diagnosis of SSc (ICD-10 code M34, excluding M34.2) between 1995 and 2015.

**Results:**

A total of 2778 incident SSc cases were identified. The mean age at time of SSc diagnosis was 56 (standard deviation 15) years and 76% were women. The overall incidence rate (per 1,000,000 person-years) of diagnosed SSc was 24.4 (95% confidence interval 23.6–25.4), with a slight increase over the study period, age- and sex-adjusted incidence rate ratio 1.02 (95% confidence interval 1.01–1.02) per 1-year increase. The 1-year all-cause mortality rate per 100 person-years decreased from 6.1 (3.1–12.2) in 1995 to 5.3 (2.5–11.1) in 2015, sex- and age-adjusted hazard ratio 0.96 (95% CI 0.94–0.98) per 1-year increase. Over the period, the average age at SSc diagnosis increased and the proportion of women decreased, whereas the burden of comorbidities increased. One fifth of all deaths were attributable to cardiovascular causes, a fourth to pulmonary diseases, and 15% were due to cancer.

**Conclusions:**

Within the last few decades, the incidence of SSc has increased and the 1-year mortality rate has decreased slightly in Denmark. Almost half of all deaths were attributable to cardiopulmonary causes.

**Electronic supplementary material:**

The online version of this article (10.1186/s41927-018-0043-6) contains supplementary material, which is available to authorized users.

## Background

Systemic sclerosis (SSc) is a heterogenic systemic autoimmune connective tissue disease characterized by humoral and cellular immune dysregulation resulting in vasculopathy and progressive fibrosis of the skin and visceral organs. SSc is also associated with substantially increased morbidity and mortality compared with the general population [1, 2].

Epidemiologically, SSc has been difficult to characterize due to its rarity, heterogeneity, imprecise date of onset and difficulty in case ascertainment. Consequently, large variability in estimates of incidence and mortality rates have been reported in prior studies [3]. Due to its rarity and imprecise date of onset, large datasets with continuous registration of diagnoses, such as nationwide administrative data, are needed to get more precise estimates of the incidence and mortality, and to investigate changes in these measures over time.

The question about trends over time is particularly interesting in the setting of SSc, as an increasing incidence of several autoimmune disorders has been noted over the last few decades [4] and there is some evidence that also the incidence of SSc may also been increasing [5, 6]. Similar, the survival over the past decades may have improved, but sparse data are available, especially on causes of death [1, 3, 7–9]. The SSc population in Denmark has been scarcely depicted [10]. In this study, we therefore aimed to estimate the incidence and the mortality rates of SSc in Denmark. We also investigated underlying causes of deaths and if these measures have changed during 1995 to 2015.

## Methods

### Data sources

All Danish citizens are given a permanent civil registration number at the time of birth or immigration. This number is used in every contact with the hospital system and at the central population registry, where all births and deaths (including dates) are registered. For the present study, we cross-linked the central population registry with the nationwide Danish National Patient Register (DNPR), which contains information on all in and out hospital visits. In brief, the DNPR is held by the Danish government in order to keep statistics of resources and needs of medical care. All medical care is tax funded and equally available to Danish citizens. For each visit to the hospital, patients are assigned one main diagnosis and any secondary diagnoses of relevance to the current hospital contact upon discharge. Financial reimbursement to the hospital departments is pending on the diagnostic codes, which makes a great incitement for correct coding. The diagnostic codes have been based on the international classification of diseases (ICD) 10th version system since 1994. Between 1978 (when the hospitalization registry was established) and 1993 the codes were based on the ICD 8th version. The ICD-9 coding system was never implemented in Denmark. The diagnostic codes utilized in this study are presented in Additional file 1. Causes of death are available from the National Causes of Death Registry. All causes of deaths (primary and any contributing causes) are registered with ICD-10 codes and assessed by the treating physicians or based on autopsy reports, if available.

### Study population

The definition of SSc in this study was based on a first-time registration of SSc using ICD-10 codes M34 except for M34.2 in the DNPR following outpatient or inpatient visits between 1995 and 2015. To ensure inclusion of only incident cases, we excluded patients with a prior diagnosis of SSc, defined as an ICD-10 code in 1994 or an ICD-8 code of 734 anytime between 1978 and 1993.

### Validation

In the DNPR, the accuracy and completeness of discharge data have been shown to be high for most diagnoses [11]. Prior epidemiological studies have used the SSc diagnosis from the DNPR under the assumption that the quality of the hospital registry data is high because patients with SSc are almost exclusively treated in specialized departments of rheumatology and dermatology [12]. Patients with SSc are usually seen at outpatient clinics where a diagnosis is typically made and registered in the DNPR. We further validated the ICD-10 diagnosis of SSc by reviewing charts at hospitals involved in the treatment of SSc patients in the Eastern Denmark. Up to 30 consecutive patients were identified in the period 2005–2010 based on DNPR data from each of three departments (two departments of rheumatology and one department of dermatology) at three different hospitals. The chosen departments were chosen to represent one highly specialized and two ordinary referral centers for connective tissue diseases reflecting the common referral process in Denmark. The study sample and period for validation was chosen based on feasibility and the sample is therefore not a completely random sample of all SSc patients in Denmark (as three departments were chosen). In total, 86 patients (26, 30, and 30 patients from each department, respectively) had available electronic charts and hereof 81 patients fulfilled the 2013 ACR/EULAR criteria for SSc, corresponding to a positive predictive value of 94%.

### Statistics

Incidence and mortality rates for SSc (by calendar year) were calculated using all Danish inhabitants who were alive per the 1st of January and who turned 18 years of age during the year. One-year mortality for SSc-cases was defined with first registration of diagnosis and censored at death or at 365 days follow-up. For the background population the 1-year mortality was defined per calendar year. Age was calculated at the 31st of December for each year. 95% confidence intervals (CIs) of the estimates were calculated under the assumption of a Poisson distribution and were used to assess if the annual rates may be fluctuating randomly. A Poisson regression analysis was used to calculate age- and sex adjusted incidence rate ratios per 1-year increase to investigate the trends over time (between 1995 and 2015). Trends in mortality rates per 1-year increase in calendar time (between 1995 and 2015) among SSc patients were also estimated using age- and sex adjusted Cox proportional hazard regression models. Standardized mortality rates (SMR), used as a mean to compare rates with the background population within selected strata, were calculated by dividing the mortality rate of SSc patients with the mortality rate in a corresponding age- and sex stratum of the background population. Test for differences in distribution of patient characteristics (within calendar bins of 1995–2001, 2002–2008, 2009–2015) was done by the ANOVA test (for continuous variables) and by the Cochran-Armitage trend test (for discrete variables), respectively. Similar, tests for differences in distributions of 1-year causes of death between the early (1995–2005) and late (2006–2015) study period was done by the Chi-squared test. Two-sided *p*-values < 0.05 were considered statistically significant for all analyses. All analyses were performed in SAS version 9.4 (Cary, NC, USA).

### Ethics

The study was approved by the Danish Data Protection Agency (ref. 2007-58-0015, int. ref. GEH-2014-018). As a retrospective registry-based study, Danish law does not require ethical approval. The chart review for the validation process was approved by the Danish Patient Safety Authority and the permission was granted based on the assumption that only physicians at a given department reviewed its own patients’ charts.

## Results

### Population characteristics

Between 1995 and 2015 a total of 2778 patients with a first-time SSc diagnoses were identified. The mean age at the time of diagnosis was around the middle of the 5th decade of life, but increased slightly throughout the study period. The proportion of women was 76% for the entire study period, but decreased slightly throughout the study period, Table 1. Diagnosed renal disease, diabetes, treated hypertension, and prior cancer at the time of the SSc diagnosis was more common in the late study period vs. the earlier periods, whereas the proportion of immigrants or prior cardiovascular diseases did not change significantly during the observational period.

**Table 1:** Demographics of SSc cases stratified by calendar time

	1995–2001	2002–2008	2009–2015	p-value
*N*	830	887	1061	
Mean age	53.7 (15.6)	55.0 (15.3)	56.1 (15.5)	0.0008
Women	652 (79%)	687 (77%)	775 (73%)	0.01
Immigrants	56 (7%)	50 (6%)	86 (8%)	0.20
Cancer	48 (6%)	51 (6%)	101 (10%)	0.001
Chronic obstructive pulmonary disease	12 (1%)	21 (2%)	48 (5%)	0.0002
Treated hypertension (defined as at least 2 antihypertensive agents)	132 (16%)	264 (30%)	471 (44%)	< 0.0001
Diabetes	25 (3%)	53 (6%)	61 (6%)	0.007
Upper gastrointestinal ulcer	47 (6%)	62 (7%)	83 (8%)	0.18
Renal disease	18 (2%)	18 (2%)	40 (4%)	0.03
Heart failure	24 (3%)	26 (3%)	34 (3%)	0.91
Prior acute myocardial infarction	28 (3%)	28 (3%)	30 (3%)	0.79
Peripheral artery disease	34 (4%)	34 (4%)	53 (5%)	0.42
Ischemic stroke	13 (2%)	23 (3%)	33 (3%)	0.10

Footnote: Continuous and categorical variables are presented as mean (standard deviation) and as frequencies (%), respectively. All diagnoses were based on ICD-8 and ICD-10 codes, except for the diabetes mellitus (based on ‘Anatomical Therapeutical Chemical’ codes of antidiabetic medication) and the hypertension diagnosis, which was based on a validated algorithm with use of at least 2 antihypertensive agents [46], Additional file 1

### Incidence

**Fig. 1:**
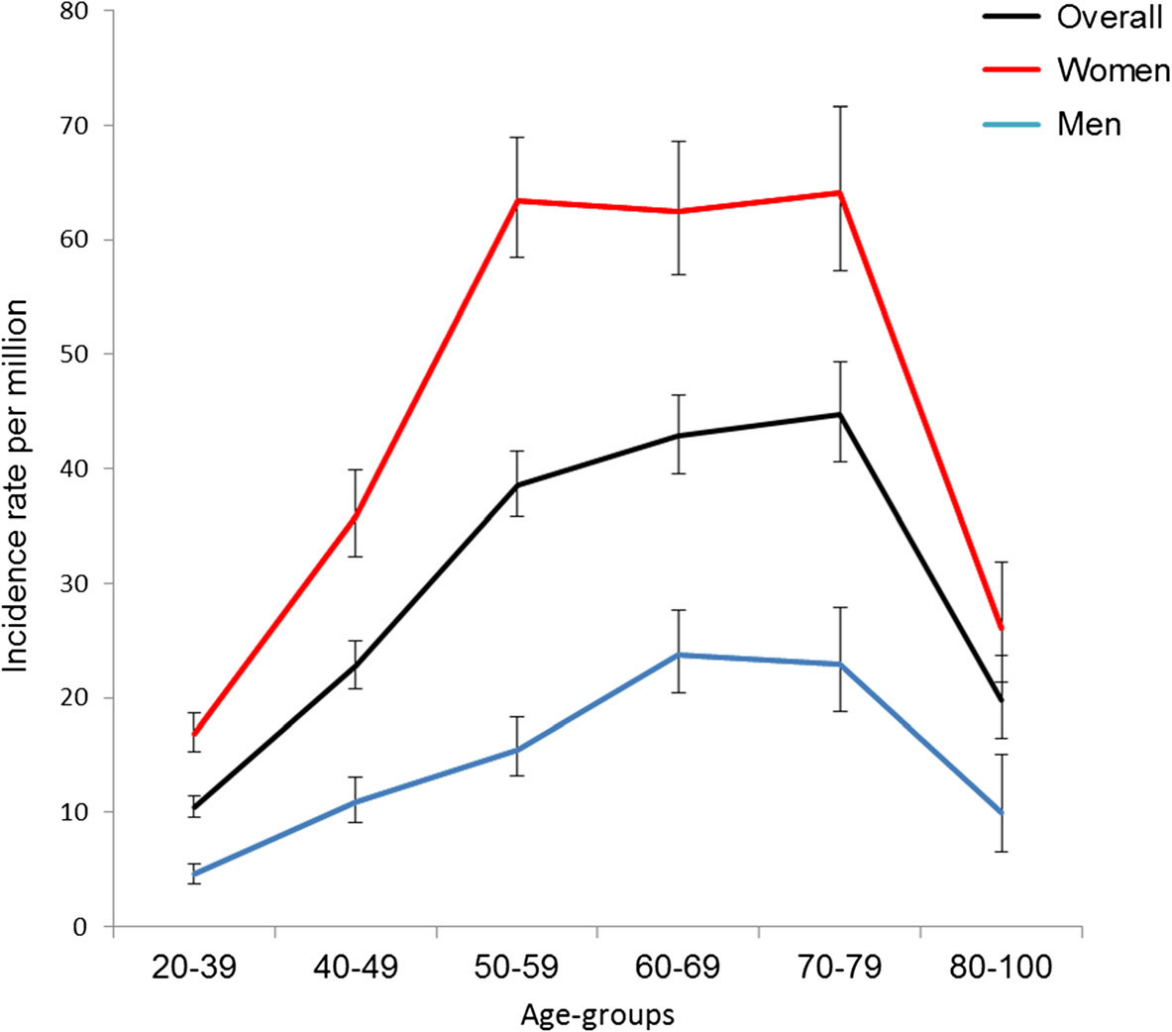
Age and sex stratified incidence rates. Incidence rates per million for men (blue) and women (red) with 95% confidence intervals

**Fig. 2:**
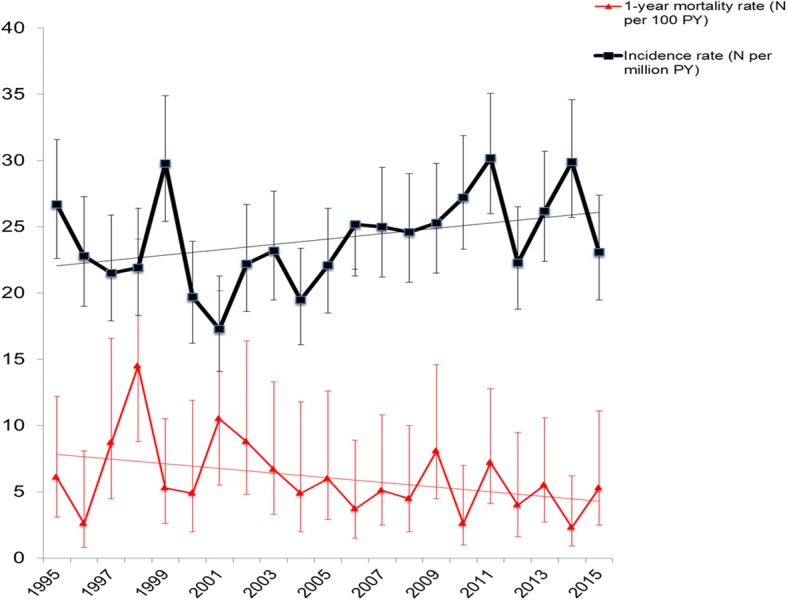
Temporal trends in overall incidence and 1-year mortality rates between 1995 and 2015. Incidence rate (black line) and mortality rate (red line) with 95% confidence intervals of estimate

**Fig. 3:**
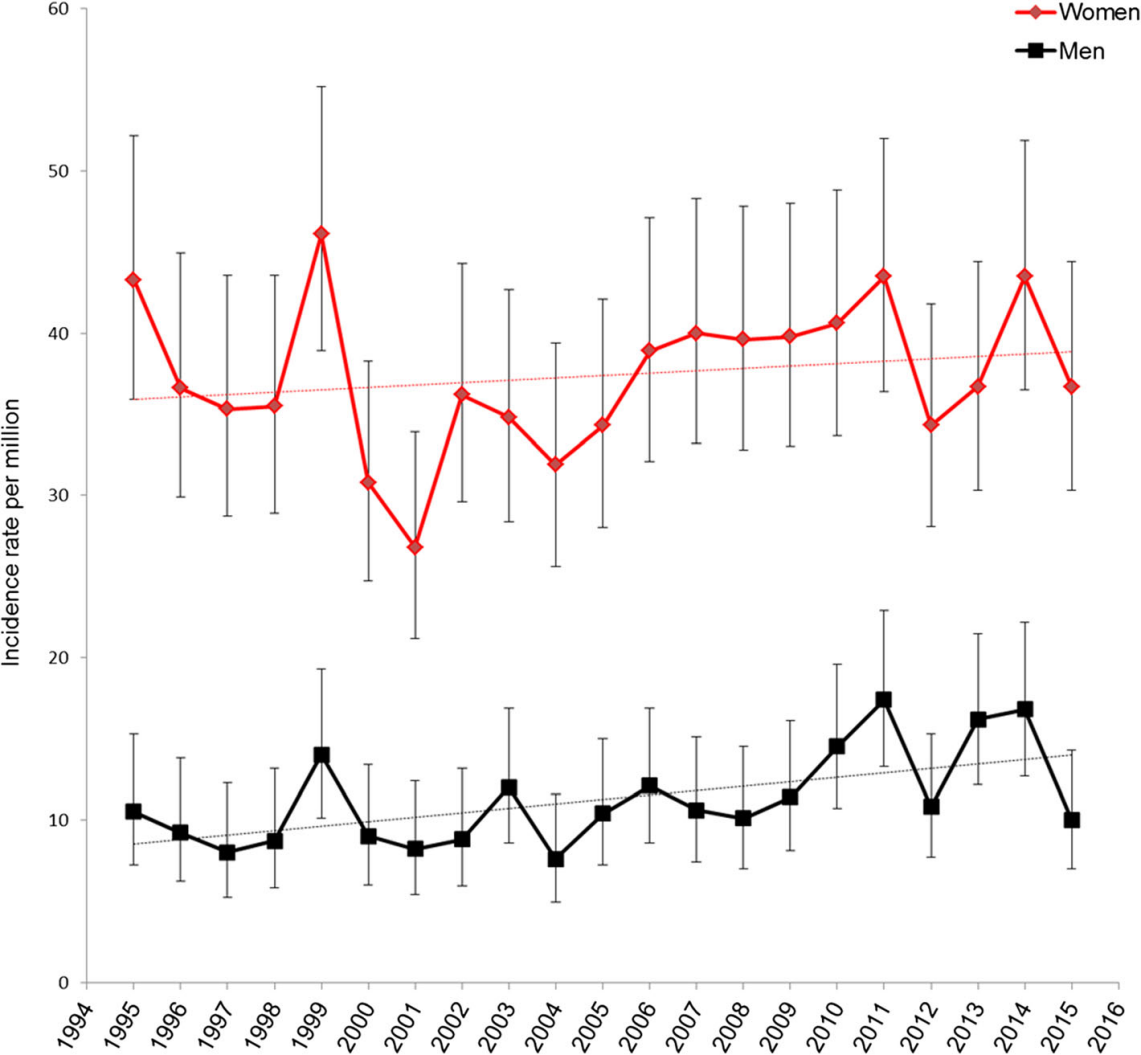
Temporal trends in sex-stratified incidence rates between 1995 and 2015. Incidence rate per million (blue and red lines) with 95% confidence intervals of estimate

Incidence rates peaked around age 60–79 years in men and at age 50–79 years in women, Fig. 1. Between 1995 and 2015, the incidence rates were slightly increasing, Fig. 2 and Additional file 2. The corresponding age- and sex-adjusted incidence rate ratio per year increase was 1.02 (95% CI 1.01–1.02). Similar trends were observed in analyses of men and women separately, Fig. 3.

### Mortality

1-year all-cause mortality was 5.8 (95% 5.0–6.8) per 100 persons among SSc patients. Compared with the background population, the sex and age-adjusted hazard ratio for 1-year mortality associated with SSc was 5.5 (95% CI 4.7–6.4). 5-year mortality rates were slightly lower than 1-year mortality rates, 3.7 (95% 3.3–4.1) per 100 person-years, but still substantially higher than the background population, Table 2. Full details of mortality rates by calendar-year for the background population and the SSc patients are available in Table 2. The mortality rates were highest in the older age-groups for both men and for women. When compared to the mortality rate of the background population, the SMR was, however, highest in the younger age-groups and declined with increasing age, Table 3.

**Table 2:** SSc mortality rates, SMR and hazard ratios

	1-year background mortality rate (per 100 PY)	1-year SSc mortality (no cases)	1-year SSc mortality rate (per 100 PY)	5-year SSc mortality (no cases)	5-mortality mortality rate (per 100 PY)	1-year SMR	1-year hazard ratio compared to background population (age and sex adjusted)
1995	1.23 (1.23–1.23)	8	6.1 (3.1–12.2)	19	3.1 (2.0–4.8)	5.0	5.3 (2.7–10.6)
1996	1.18 (1.17–1.19)	≤3	2.6 (0.8–8.1)	24	4.6 (3.1–6.8)	2.2	2.1 (0.7–6.4)
1997	1.16 (1.15–1.17)	9	8.7 (4.5–16.6)	24	4.9 (3.3–7.4)	7.5	8.3 (4.4–16.0)
1998	1.12 (1.11–1.13)	15	14.5 (8.8–24.1)	20	4.1 (2.6–6.3)	13.0	14.3 (8.6–23.7)
1999	1.13 (1.11–1.14)	8	5.3 (2.6–10.5)	28	4.0 (2.8–5.8)	4.7	5.2 (2.6–10.4)
2000	1.09 (1.08–1.10)	5	4.9 (2.1–11.9)	13	2.7 (1.6–4.7)	4.5	5.3 (2.2–12.6)
2001	1.10 (1.08–1.10)	9	10.5 (5.5–20.2)	23	6.1 (4.0–9.1)	9.6	9.6 (5.0–18.3)
2002	1.10 (1.09–1.10)	10	8.8 (4.8–16.4)	21	4.0 (2.6–6.1)	8.0	7.6 (4.1–14.1)
2003	1.07 (1.06–1.07)	8	6.7 (3.3–13.3)	27	5.0 (3.4–7.2)	6.3	6.5 (3.3–13.0)
2004	1.03 (1.02–1.03)	5	4.9 (2.0–11.8)	12	2.4 (1.4–4.3)	4.8	5.2 (2.2–12.4)
2005	1.00 (0.99–1.01)	7	6.0 (2.9–12.6)	26	4.9 (3.3–7.2)	6	5.6 (2.7–11.8)
2006	1.00 (1.00–1.01)	5	3.7 (1.5–8.9)	16	2.5 (1.5–4.0)	3.7	4.1 (1.7–9.8)
2007	1.00 (0.99–1.00)	7	5.1 (2.5–10.8)	18	2.8 (1.8–4.4)	5.1	6.2 (3.0–13.0)
2008	0.97 (0.96–0.98)	6	4.5 (2.0–10.0)	19	3.0 (1.9–4.7)	4.6	4.7 (2.1–10.4)
2009	0.97 (0.96–0.98)	11	8.1 (4.5–14.6)	23	3.6 (2.4–5.4)	8.4	8.2 (4.5–14.7)
2010	0.95 (0.94–0.96)	4	2.6 (1.0–7.0)	23	3.2 (2.1–4.8)	2.7	2.8 (1.1–7.4)
2011	0.91 (0.91–0.92)	12	7.2 (4.1–12.8)	25	3.2 (2.2–4.7)	7.9	8.9 (5.1–15.8)
2012	0.90 (0.89–0.91)	5	3.6 (1.6–9.5)	22	NA	4.0	4.8 (2.0–11.5)
2013	0.90 (0.89–0.91)	8	5.5 (2.7–11.0)	22	NA	6.1	6.0 (3.0–12.1)
2014	0.88 (0.87–0.89)	4	2.3 (0.9–6.2)	12	NA	2.6	3.0 (1.1–8.0)
2015	0.90 (0.89–9.90)	7	5.3 (2.5–6.2)	10	NA	5.9	5.6 (2.7–11.8)
Overall	1.02 (1.02–1.03)	NA	5.8 (5.0–6.8)	427	3.7 (3.3–4.1)	5.7	5.5 (4.7–6.4)

Footnote: SMR denotes standardized mortality rate (i.e., mortality rate in SSc patients divided by mortality rate in background population). Mortality rates and hazard ratio are presented with 95% CIs

**Table 3:** 1-year mortality rates in SSc patients stratified by age and sex, compared with the background population

	Overall				Men			Women		
Age groups	Background mortality rate	Case mortality rate	SMR	Number of deaths in SSc group	Background mortality rate	Case mortality rate	SMR	Background mortality rate	Case mortality rate	SMR
20–39	0.05 (0.05–0.05)	0.63 (0.20–1.95)	13.4	≤3	0.06 (0.06–0.06)	0.93 (0.13–6.57)	14.9	0.03 (0.03–0.03)	0.54 (0.14–2.17)	18
40–49	0.17 (0.17–0.18)	1.97 (1.02–3.78)	11.6	9	0.21 (0.20–0.21)	3.53 (1.32–9.40)	17.0	0.14 (0.14–0.14)	1.45 (0.60–3.49)	10.7
50–59	0.47 (0.47–0.48)	3.19 (2.10–4.85)	6.8	22	0.56 (0.55–0.56)	6.37 (3.31–12.24)	11.4	0.38 (0.38–0.39)	2.38 (1.38–4.09)	6.2
60–69	1.19 (1.18–1.19)	5.99 (4.30–8.35)	5.0	35	1.39 (1.39–1.40)	6.05 (3.25–11.24)	4.4	0.98 (0.97–0.98)	5.97 (4.04–8.84)	6.1
70–79	3.16 (3.15–3.17)	14.63 (11.24–19.06)	4.6	55	3.72 (3.70–3.74)	21.90 (13.97–34.34)	5.9	2.67 (2.65–2,68)	12.45 (8.98–17.27)	4.7
80–100	9.45 (9.43–9.48)	33.15 (23.44–46.88)	3.5	32	9.74 (9.70–9.78)	64.61 (34.76–120.08)	6.6	9.27 (9.24–9.30)	27–14 (17.87–41.22)	2.9

Footnote: Mortality rates are expressed per 100 person-years. SMR denotes standardized mortality rate (i.e., mortality rate in SSc patients divided by mortality rate in background population)

**Fig. 4:**
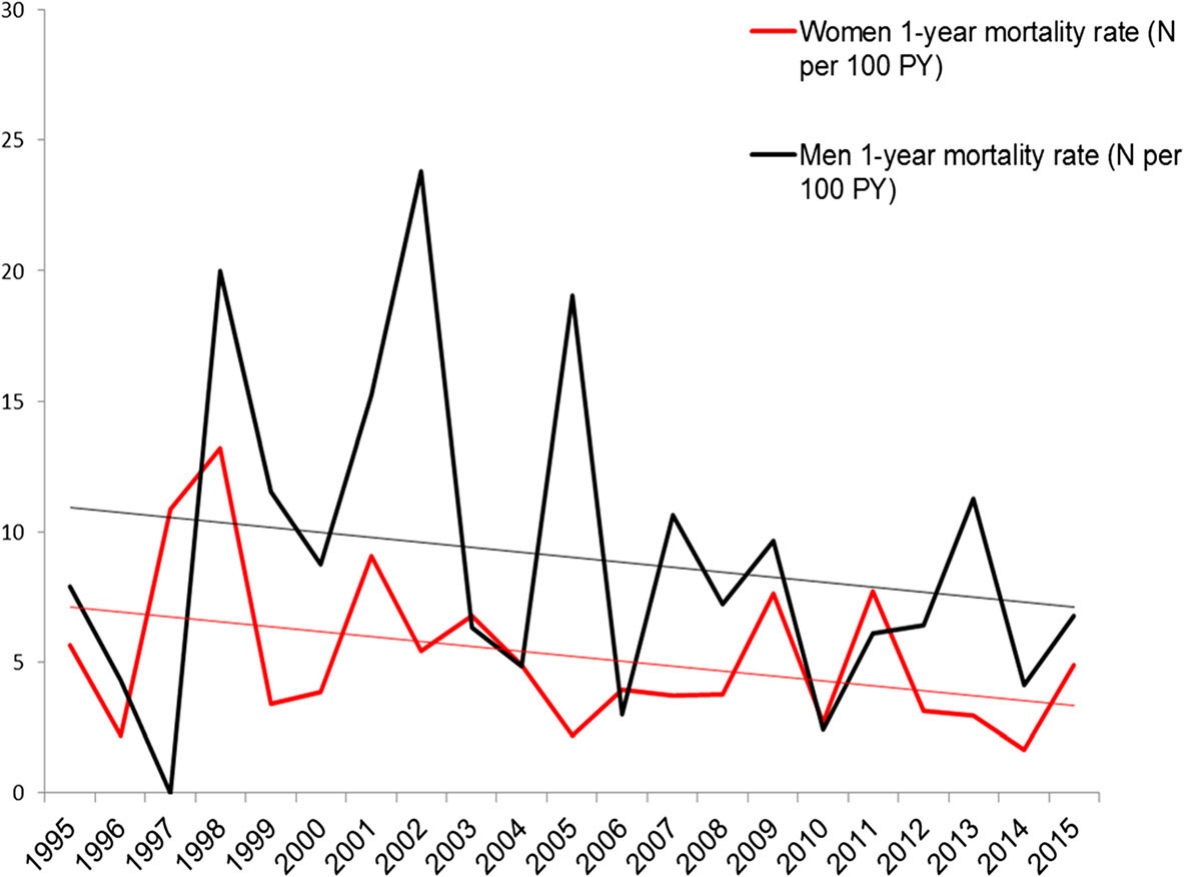
Temporal trends in sex-stratified mortality rates between 1995 and 2015. 1-year mortality rates for men and women

As illustrated in Fig. 2 (and in Table 2), the all-cause 1-year mortality rate per 100 person-years decreased from 1995 to 2015 with a sex- and age-adjusted annual hazard ratio of 0.96 (95% CI 0.94–0.98) per 1-year increase within the SSc population. Similar trends were noted for both women and men, Fig. 4. Causes of death for all SSc patients dying at any time during the observational period are given in Table 4. For the 1-year mortality endpoint specifically, the primary cause of death was in 22% attributed to cardiovascular disorders, in 17% to pulmonary causes, in 9% to cancer, and in 22% to musculoskeletal causes (the majority being SSc itself without further specification). These distributions did not differ for the early (1995–2005) and late (2005–2015) period (all *p* > 0.25), except for the musculoskeletal causes which were statistically more common in the early vs. late period (29% vs. 7%, *p* = 0.0007).

**Table 4:** Primary causes of death from 1995 to 2015

A00-B99	Certain infectious and parasitic diseases	5%
A31	Sepsis, other	4%
C00-D48	Neoplasms	15%
C34	Malignant neoplasm of bronchus and lung	4%
I00-I99	Diseases of the circulatory system	19%
I60-I69	Cerebrovascular diseases	3%
I20-I25	Ischaemic heart diseases	4%
I50	Heart failure	4%
J00-J99	Diseases of the respiratory system	24%
J96	Respiratory failure, not elsewhere classified	13%
J09-J18	Influenza and pneumonia	7%
K00-K93	Diseases of the digestive system	3%
M00-M99	Diseases of the musculoskeletal system and connective tissue	9%
M34	Systemic sclerosis	8%
R00-R99	Symptoms, signs and abnormal clinical and laboratory findings, not elsewhere classified	16%

Footnote: Registered primary causes of death among SSc patients dying at any time during the observational period. Only groups ≥3% are specified (due to Danish laws about identifiable data)

## Discussion

Using administrative registries of the whole population, we have herein described the incidence and the mortality of SSc in Denmark over the last couple of decades. In keeping with former epidemiologic studies [3, 13], we found SSc to be more common in women than in men with an overall woman: men ratio of approximately 4:1. The mean age of diagnosis was around the middle of the 5th decade of life, but was slightly increasing between 1995 and 2015. Our main observation includes a small, but steady increase in incidence rates throughout the study period. Moreover, incidence rates increased gradually with advancing age until the age of 60–79 years for men and of 50–79 years for women. These observations are consistent with the literature [5, 14]. Our second principle finding was that mortality rates decreased slightly during the study period. Still, SSc patients remained to have a substantially increased risk of death, when compared to the general population at the end of the study period.

### Incidence

Our study extends other studies that have shown an increase in the incidence rate of SSc over recent times. However, large discrepancies have been noted among regions and methodological concerns have been raised. Earlier and smaller studies have reported lower incidence rates in the Northern Europe (Finland, Iceland, Norway, England) [15–18], as compared to the Southern Europe (Spain, Greece, France, Italy) [5, 19–22]. This has led to speculations of a north-south European gradient of SSc. A recent study in Sweden [23], however, has shown substantially higher rates, with annual incidence rate estimates as high as 19 per million inhabitants, which does not support the existence of such a gradient. Our study revealed higher incidence rates than other northern European estimates, with an overall incidence rate of 24 per million adults. However, our study represents the only newer nationwide European study, whilst almost all other studies have been based on selected cohorts. Studies in certain geographical clusters in Italy, England and Australia [3, 24] have pointed towards unexpectedly high cumulated incidence rates albeit the actual number of patients has been low. When comparing studies from other continents over time, recent studies have also shown an increasing trend in incidence and prevalence rates e.g. in Australia [25] (22.8 incident cases per million) and the U.S. [14] (19.3 incident cases per million). In contrast, low rates have been estimated in Taiwan (10.9 incident cases per million) [26].

Due to the absence of common diagnostic criteria until the eighties and only recent inclusion of milder and possibly earlier variants of disease, prior estimates of incidence should be interpreted with caution. Some of the considerable differences across time, space and ethnic diversity can be speculated to be due to, in part at least, the methodology applied in the respective studies. After the introduction of the ACR/EULAR 2013 criteria some reports have cited a 30–40% increase in incidence and prevalence rates [23], while others have reported an increase of 26% in the number of patients fulfilling the disease definition in comparison with the 1980 ARA criteria [27, 28]. Our study found a substantially smaller increase over the time period than those prior estimates albeit an increased incidence proportion in the latter timespan from 2008 to 2015 compared to the first from 1995 to 2002. The latter could perhaps also be explained by e.g. increasing use of serological markers or capillaroscopy leading to a diagnosis of early and mild disease. In contrast to what may be expected with more sensitive diagnostic criteria (where possibly earlier and milder diseases may be included) our mean age increased throughout the study period. Similarly, the demographic characteristics yielded a higher diagnosed comorbidity burden within recent years. The etiology behind this may be multifactorial and in part due to an ageing population, more adverse lifestyle manifestations, earlier diagnosis, lower threshold for treatment, better access to screening programs and specialized care. The increasing comorbidity however is also an argument against that less sick people are included with the new diagnostic criteria. This could suggest that the observed increase in incidence may, at least in part, represent a true increase rather than the substantial increases resulting merely from methodological differences. Yet, the varying diagnostic criteria used over time and the rarity of the disease prohibit any conclusions on this matter (as discussed in detail below under the limitations section).

### Mortality

The increased morbidity and mortality observed in SSc is well-known, and several meta-analyses have reported a high overall SMR for SSc ≥ 3 [1, 2, 8]. However, there are several studies from the 1960’s and onwards indicating higher survival rates in recent cohorts compared with older ones [14, 29–31]. These findings are somewhat consistent with our study, with falling 1-year mortality rates from 1995 to 2015, although the observed decline was rather small. The SMR and hazard ratio were at the higher end of earlier reported figures with an overall SMR of 5.7, as compared with prior pooled SMR estimates of 3–4 [1, 2, 10, 32]. Perhaps, this difference could be explained by our study being a nationwide cohort study and thus more inclusive not limited by sample size or certain age-groups. Comparing age-groups, we expectedly (and in accordance with prior studies [33–36] found that older age of diagnosis was linked to higher mortality rates. SMR, however, peaked in younger age-groups and was not significantly different in males and females. Such findings were also noted in a prior Danish cohort [10]. Information on the modifying role of age at onset in outcome has been inconsistent, but our findings with higher mortality rates in the elderly may be explained by increased occurrence of comorbidities and the reduced life expectancy in this age group. Finally, our SMR was calculated from 1-year mortality rates, which would expectedly be higher than e.g. long-term mortality rates.

Although the lowered mortality rates within the more recent years, in theory, could be explained by lead time bias (meaning that an earlier diagnosis of e.g. mild or early limited cutaneous SSc falsely appears to prolong survival), several things suggest otherwise. First, the mean age and comorbidity burden increased throughout the years in our cohort. Secondly, the main causes of death did not seem to change over the study period (although we admittedly had low power to detect smaller differences). One possibility underlying the increased survival could be related to more aggressive management of e.g. hypertension and other comorbidities, possibly consistent with our observations of more treated patients in the later study period. Currently available treatments for SSc related internal organ involvement e.g. sclerodermal renal crisis, pulmonary hypertension, and more aggressive strategies for interstitial lung disease have also been hypothesized to have changed the pattern of causes of mortality in SSc [31, 37, 38]. We did not observe such pattern changes during our 20-year study period, although we observed a lower proportion of deaths directly attributable to SSc in the late vs. early period. Whether this may be a true finding or changes in registration practice, is, however, unknown. Notably, our study period was limited to 1995–2015 and did not include the calendar time before the introduction of recent treatment approaches. Cardiopulmonary manifestations are currently believed to be among the leading causes of death in SSc [8, 39, 40], which is also supported by our study data. Still, nearly half of all deaths were due to other causes, including approximately one seventh of all deaths being primarily attributed to a cancer diagnosis, underlining the need for a consistent focus on non-SSc comorbidity. The association between SSc and cancer is well recognized and an area warranting more research [41, 42].

### Study strengths and weaknesses

In general, Denmark is well-suited for epidemiological studies of rare diseases, as the population is stable and homogeneous, and as the Danish registries, including the hospitalization registries, are accurate and complete [43, 44]. Considering the findings of the validation process and the high positive predictive value (94%) of the diagnosis in the DNPR, this report is submitted on the assumption that the SSc diagnosis is valid and accurate. However, the validation process was not based on a completely random selection of patient files due to the infeasibility of doing so (restrained by Danish laws). The principle strength of this study is the unselected nature of the population. A major limitation is the lack of validation of causes of death which were based on clinicians’ adjudication. Furthermore, as the specific patient context was not available via the National Causes of Death Registry, it was not possible to classify cause of death and organ involvement as SSc or non-SSc related.

It would have further strengthened the study if it had been possible to discriminate lcSSc from dcSSc, but this was unfortunately not feasible due to the lack of detail in the ICD10 diagnostic coding. Moreover, for patients with prevalent SSc immigrating to Denmark, these could be mis-classified as incident cases in our study. However, the proportion of patients being immigrants was low and not significantly different in early and later study periods, reducing the likelihood that our period results were influenced hereby.

## Conclusion

The incidence rate of SSc was approximately 24 per million inhabitants in Denmark, which is somewhat greater than that reported in many prior studies. Further, the incidence rates increased slightly from 1995 to 2015. Over the same period, 1-year mortality rates declined. The main causes of death did not seem to change over the study period although the demographic characteristics of patients with newly diagnosed SSc changed, i.e. patients were older and had more comorbidity at time of diagnosis in the later vs. earlier years. As a seventh of all patients died from cancer, more attention may be warranted on this disease in SSc patients. Further epidemiological studies of SSc based on uniform classification criteria over time are warranted to confirm our hypothesis-generating findings.

## Additional files

Additional file 1: Variables and sources. Diagnoses (ICD-8 and ICD-10) and medication (ATC, Anatomical Therapeutical Chemical) codes used. (DOCX 20 kb)

Additional file 2: Overall incidence rates, mean age at diagnosis and proportion of women in 1995–2015. Description: The table depicts data from 1995 to 2015 including number of cases per year, observation time (total person years), incidence rate per million, mean age at onset, and proportion of women (%). (DOCX 20 kb)

## Abbreviations

ACR: American College of Rheumatology
DNPR: Danish National Patient Registry
EULAR: European League against Rheumatism
ICD: international classification of diseases
SMR: standardized mortality rates
SSc: systemic sclerosis
