# Supplementary material for: Trends in incidence, mortality, and causes of death associated with systemic sclerosis in Denmark between 1995 and 2015: a nationwide cohort study
Source: BMC Rheumatol. 2018 Dec 7;2:36. doi: 10.1186/s41927-018-0043-6 (PMC6390621; doi:10.1186/s41927-018-0043-6)
Supplement: Supplementary file 1 — Variables and sources. Diagnoses (ICD-8 and ICD-10) and medication (ATC, Anatomical Therapeutical Chemical) codes used. (DOCX 20 kb) [file 41927_2018_43_MOESM1_ESM.docx]

**Additional File 1.** Variables and sources. Diagnoses (ICD-8 and ICD-10) and medication (ATC, Anatomical Therapeutical Chemical) codes used.

| **Disease/condition** | **Relevant ICD8 or ICD10 diagnostic codes (DNPR) or ATC codes (Dansh Register of Medicinal Product Statistics)** |
| --- | --- |
| Cancer | ICD-8: 140-200  ICD-10: C00-C97 |
| Chronic obstructive pulmonary disease | ICD-8: 491-492.  ICD-10: J44. |
| Treated hypertension (defined as at least 2 antihypertensive agents) | ATC: C02A, C02B, C02C, C02DA, C02L, C02DB, C02DD, C02DG, C03A, C03B, C03D, C03E, C03X, C04, C05, C07A, C07B, C07C, C07D, C07F, C08, C08G, C09AA, C09BA, C09BB, C09CA, C09DA, C09DB, C09XA02, C09CA52 |
| Diabetes | ATC: A10  . |
| Upper gastrointestinal ulcer | ICD-8: 531-535  ICD-10: K25-K27, K29 |
| Renal disease | ICD-8: 403-404, 581-584, 75310, 75311, 75319, 59009, 59320, 25002, 40039  ICD-10: N02-N12, N14, N18, N19, N26, Q61, N158-N164, N168, Q613, E102, E112, E132, E142, I120, M321B |
| Heart failure | ICD-8: 425, 4270, 4271  ICD-10: I42, I50, I110 |
| Prior acute myocardial infarction | ICD-8: 410.  ICD-10: I21, I22 |
| Peripheral artery disease | ICD-8: 440  ICD-10: I70, I74 |
| Ischemic stroke | ICD-8: 433  ICD-10: I63, I64 |
