# Supplementary material for: Trends in incidence, mortality, and causes of death associated with systemic sclerosis in Denmark between 1995 and 2015: a nationwide cohort study
Source: BMC Rheumatol. 2018 Dec 7;2:36. doi: 10.1186/s41927-018-0043-6 (PMC6390621; doi:10.1186/s41927-018-0043-6)
Supplement: Supplementary file 2 — Overall incidence rates, mean age at diagnosis and proportion of women in 1995–2015. Description: The table depicts data from 1995 to 2015 including number of cases per year, observation time (total person years), incidence rate per million, mean age at onset, and proportion of women (%). (DOCX 20 kb) [file 41927_2018_43_MOESM2_ESM.docx]

**Additional File 2. Overall incidence rates, mean age at diagnosis and proportion of women in 1995-2015.**

|  | | | |  |  |
| --- | --- | --- | --- | --- | --- |
|  |  |  |  |  |  |
|  | N cases | Total person-years | Incidence rate per million (95% CI) | Mean age at onset (min-max) | Proportion women (%) |
|  |  |  |  |  |  |
| 1995 | 136 | 5.085.353 | 26.7 (22.6-31.6) | 55 (20-86) | 80 |
| 1996 | 117 | 5.134.300 | 22.8 (19.0-27.3) | 55 (21-90) | 79 |
| 1997 | 111 | 5.157.970 | 21.5 (17.9-25.9) | 55 (18-85) | 81 |
| 1998 | 114 | 5.196.716 | 21.9 (18.3-26.4) | 53 (18-84) | 80 |
| 1999 | 156 | 5.231.443 | 29.8 (25.4-34.9) | 54 (22-87) | 76 |
| 2000 | 104 | 5.282.740 | 19.7 (16.2-23.9) | 52 (18-83) | 77 |
| 2001 | 92 | 5.304.499 | 17.3 (14.1-21.3) | 54 (19-90) | 76 |
| 2002 | 119 | 5.341.755 | 22.2 (18.6-26.7) | 58 (21-88) | 80 |
| 2003 | 125 | 5.381.449 | 23.2 (19.5-27.7) | 55 (23-93) | 74 |
| 2004 | 106 | 5.439.001 | 19.5 (16.1-23.4) | 55 (22-97) | 80 |
| 2005 | 121 | 5.467.875 | 22.1 (18.5-26.4) | 57 (19-86) | 76 |
| 2006 | 139 | 5.514.347 | 25.2 (21.3-21.8) | 55 (21-87) | 76 |
| 2007 | 139 | 5.559.583 | 25.0 (21.2-29.5) | 52 (18-85) | 78 |
| 2008 | 138 | 5.620.506 | 24.6 (20.8-29.0) | 57 (19-87) | 79 |
| 2009 | 143 | 5.646.869 | 25.3 (21.5-29.8) | 57 (18-94) | 77 |
| 2010 | 155 | 5.689.390 | 27.2 (23.3-31.9) | 57 (19-90) | 73 |
| 2011 | 173 | 5.727.783 | 30.2 (26.0-35.1) | 55 (18-98) | 71 |
| 2012 | 129 | 5.782.198 | 22.3 (18.8-26.5) | 56 (18-81) | 75 |
| 2013 | 152 | 5.798.987 | 26.2 (22.4-30.7) | 57 (18-91) | 68 |
| 2014 | 174 | 5.827.941 | 29.9 (25.7-34.6) | 56 (19-89) | 71 |
| 2015 | 135 | 5.852.478 | 23.1 (19.5-27.4) | 59 (19-89) | 78 |
| Overall | 2778 | 115.043.183 | 24.4 (23.6-25.4) | 56 (18-90) | 76 |

Footnote: Incidence rates are presented with 95% CIs, mean age with range, and the proportion of women as percentages.
